# Clinical, serological and echocardiographic examination of healthy field dogs before and after vaccination with a commercial tetravalent leptospirosis vaccine

**DOI:** 10.1186/s12917-017-1056-x

**Published:** 2017-05-25

**Authors:** Andrea M. Spiri, Sabrina Rodriguez-Campos, José M. Matos, Tony M. Glaus, Barbara Riond, Claudia E. Reusch, Regina Hofmann-Lehmann, Barbara Willi

**Affiliations:** 1 Clinical Laboratory, Vetsuisse Faculty, University of Zurich, Winterthurerstr. 260, 8057 Zurich, Switzerland; 2 Center for Clinical Studies, Vetsuisse Faculty, University of Zurich, Winterthurerstr. 260, 8057 Zurich, Switzerland; 3 Clinic for Small Animal Internal Medicine, Vetsuisse Faculty, University of Zurich, Winterthurerstr. 260, 8057 Zurich, Switzerland; 4 Institute of Veterinary Bacteriology, Vetsuisse Faculty, University of Bern, Länggassstr. 122, 3001 Bern, Switzerland

**Keywords:** Nobivac® Lepto 6, *Leptospira*, Tetravalent vaccine, Vaccination, Seroconversion, Microscopic agglutination test, Adverse events

## Abstract

**Background:**

Leptospirosis is a re-emerging bacterial zoonosis caused by spirochetes of the genus *Leptospira.* Severe disease has been reported in dogs in Europe despite vaccination with bivalent *Leptospira* vaccines. Recently, a tetravalent canine *Leptospira* vaccine (Nobivac® L4) was licenced in Europe. The goal of this study was to investigate clinical signs, microscopic agglutination test (MAT) titres, haematology, blood biochemistry, cardiac (c) Troponin I levels and echocardiography before and after vaccination with this tetravalent vaccine. Forty-eight healthy dogs were prospectively enrolled and vaccinated twice, 3–4 weeks apart (T0 and T1). Before vaccination (T0) and 16–31 days after the second vaccination (T2), MAT (*n* = 48), haematology (*n* = 48), blood biochemistry (*n* = 36) and cTroponin I measurements (*n* = 29) were performed, and MAT was repeated 347–413 days after the second vaccination (T3, *n* = 44). Echocardiography was performed before the first and second vaccination (T0 and T1, *n* = 24).

**Results:**

Mild and transient clinical signs within 5 days following the first and second vaccination occurred in 23% and 10% of the dogs, respectively. Before the first vaccination (T0), all dogs showed negative MAT titres for the tested serovars except for Canicola (50% with titres 100–400). At T2, positive MAT titres to the serovars Canicola (100%), Australis (89%), Grippotyphosa (86%), Bratislava (60%), Autumnalis (58%), Copenhageni (42%), Pomona (12%), Pyrogenes (8%) and Icterohaemorrhagiae (2%) were found. Median to high titres (≥ 400) were most common to the serovar Canicola (92%) and less common to the serovars Australis (41%), Grippotyphosa (21%), Bratislava (12%), Autumnalis (4%), Pyrogenes (4%) and Pomona (2%). At T3, positive MAT titres (titre range: 100–400) were found in 2–18% of the dogs to serovars of the vaccine serogroups and in 2–18% of the dogs to the non-vaccine serovars Pomona, Autumnalis, Pyrogenes and Ballum. Haematology, blood biochemistry, cTroponin I levels and echocardiography results did not change significantly following vaccination.

**Conclusions:**

Clinical signs following vaccination with Nobivac® L4 were transient and mild in all cases. Seroconversion differed considerably among individual dogs and among the vaccine serogroups.

**Electronic supplementary material:**

The online version of this article (doi:10.1186/s12917-017-1056-x) contains supplementary material, which is available to authorized users.

## Background

Leptospirosis is a re-emerging bacterial zoonosis of global importance caused by spirochetes of the genus *Leptospira* [1]. Dogs can be infected with a wide range of pathogenic *Leptospira* serovars and shed the leptospires in their urine during acute infection [2]. Leptospires can survive for months in water and moist soil [3], and indirect transmission through contact with contaminated soil or water is thought to play an important role in the epidemiology of leptospirosis.

In Europe, canine leptospirosis in dogs has gained increasing attention in the last several years [4]. Following infection with pathogenic leptospires, dogs can develop a severe, multi-systemic disease associated with high mortality [2]. Besides this severe course of infection, mild clinical signs and asymptomatic infections have been documented. Acute leptospirosis in dogs is mainly associated with acute kidney or liver injury and respiratory signs associated with pulmonary haemorrhage [5]. Furthermore, myocarditis has been documented in humans with leptospirosis and has also been suspected in infected dogs [6, 7]. The clinical manifestation of leptospirosis depends not only on the causative serovar but also on host-specific factors such as age and the immune status of the dog [8].

The most commonly used diagnostic test for leptospirosis in dogs is the demonstration of antibodies in the microscopic agglutination test (MAT). Serial dilutions of patient sera are thereby incubated with a selection of pathogenic *Leptospira* serovars, and agglutination of the organisms is assessed by darkfield microscopy. Cross-reactivity among serovars only allows for a serogroup-specific diagnosis, and positive titres following vaccination, previous exposure and during chronic asymptomatic infection complicate the interpretation of the results [2]. Generally, the serovar that gives the highest titre is regarded as the infecting serogroup or serovar, although this diagnosis is, at best, presumptive [9]. Post-vaccination titres have been assessed in small groups of dogs following vaccination with bivalent and tetravalent vaccines [10–12]. These studies revealed that titres usually decline by 4 months after vaccination, although persistently high titres have been documented which have been explained by natural exposure to *Leptospira* field serovars [10]. PCR can be used as an alternative diagnostic method to detect leptospiral DNA in biological samples such as blood, urine or tissue. However, leptospires can only transiently be detected in the blood and urine of infected animals [13], and antibiotic therapy is thought to clear bacteraemia and leptospiruria [14]. The detection of the agents in biological samples by bacterial culture is a definitive proof of infection [2], but leptospires are fastidious organisms requiring special culture media, and they grow very slowly after 2 to up to 24 weeks in culture, making this diagnostic procedure impractical in a clinical setting.

Despite the introduction of efficient bivalent *Leptospira* bacterin vaccines some decades ago [15], clinical leptospirosis has recently been documented among numerous vaccinated dogs in Europe [16]. The currently licenced bivalent *Leptospira* vaccines in Europe contain serovars belonging to the serogroups Canicola and Icterohaemorrhagiae. These vaccines have been shown to prevent leptospirosis caused by serovars belonging to these two serogroups, but the vaccines induce only partial or no immunity to heterologous serogroups [17–19]. Canicola and Icterohaemorrhagiae were previously thought to be the main serogroups causing clinical leptospirosis in dogs. Recent seroepidemiological studies revealed, however, that dogs are susceptible to a broad range of serovars. In Europe, antibodies against the serogroups Icterohaemorrhagiae, Canicola, Grippotyphosa, Pomona, Australis and Sejroe have most commonly been documented in clinically healthy and diseased dogs [4].

In 2012, a new canine tetravalent inactivated *Leptospira* vaccine (Nobivac® L4, in Switzerland licenced as Nobivac® Lepto 6, MSD Animal Health GmbH, Luzern, Switzerland) that contains antigens from serovars from four different serogroups, including Canicola, Icterohaemorrhagiae, Australis and Grippotyphosa was licenced in Europe [20, 21]. Because serology represents the mainstay in the diagnosis of leptospirosis in dogs, the serological response following vaccination can interfere with the interpretation of MAT results in suspect clinical cases [22]. So far, no data on the serological response following vaccination with this novel tetravalent vaccine have been published. Furthermore, recent pharmacovigilance reports from Germany and Switzerland suggest that adverse events after vaccination with *Leptospira* vaccines have significantly increased since 2013 and 2014, respectively, following the introduction of novel, multivalent *Leptospira* vaccines in these countries [23, 24]. The authors of the reports speculated that the increasing number of *Leptospira* serovars included in these vaccines could result in higher antigenicity of the vaccines. However, studies investigating the incidence of adverse events following the vaccination of dogs did not reveal a significant increase in hypersensitivity reactions following *Leptospira* vaccination when compared with other vaccines [25, 26]. Finally, there have been recent anecdotal reports describing cardiac abnormalities suspicious of acute myocarditis after vaccination (S. Jenni, personal communication). Specifically, at the second vaccination, new heart murmurs had been auscultated that had not been present at the first vaccination. Subsequent echocardiographic examinations reportedly had revealed left ventricular dilation and hypocontractility with mitral annulus dilation and associated mitral insufficiency as the explanation for the murmur.

The goal of the present study was to evaluate the serological response of dogs after vaccination with a commercially available, inactivated, tetravalent *Leptospira* vaccine and to investigate whether the dogs showed clinical signs and alterations in haematology, blood biochemistry and echocardiography following vaccination.

## Methods

### Study design, sample and data collection and sample processing

This monocentric, longitudinal, prospective study was performed at the Clinic for Small Animal Internal Medicine, University of Zurich, between August 2013 and May 2015. Dogs were prospectively enrolled within the study when they met the following inclusion criteria: older than 8 weeks of age, judged clinically healthy by the owner, in good general health according to clinical examination, no vaccination within 4 weeks and no medical therapy within 2 months prior to study enrolment, with the exception of antiparasitic treatment. The study design and number of dogs that received each procedure are shown in Table 1. The day of the first vaccination was defined as T0. All dogs were vaccinated twice with a commercial tetravalent leptospirosis vaccine (Nobivac® L4, in Switzerland licenced as Nobivac® Lepto 6, MSD Animal Health GmbH) 3–4 weeks apart (T0 and T1). Additional vaccine components licenced for concurrent administration were applied as required (Table 1). Blood samples were collected by venipuncture before the first (T0) and second vaccination (T1) and 41–54 days (T2) and 368–434 days after the first vaccination (T3). Blood samples were processed or frozen at −80 °C within 4 h of collection. A medical history and clinical examination were performed at each presentation by a veterinary surgeon (AS) or a board-certified internal medicine specialist (BW). Echocardiography was performed before the first (T0) and second vaccination (T1) by board-certified cardiologists (JM, TG) at the division of cardiology, Vetsuisse Faculty, University of Zurich. Adverse vaccine events were suspected when clinical signs occurred within 5 days of vaccination and belonged to one of the following groups: lethargy or inappetence, local reaction at the injection site (signs of tenderness or pain, cutaneous swelling or mass, lameness in a limb close to the injection site), non-specific signs (periuria, diarrhoea, vomiting) or a hypersensitivity reaction (swelling of the face, hives or bumps on the face or body, hypersalivation, tachypnoea, or respiratory distress). Data on adverse vaccine events were collected from the medical history and clinical examination performed at each presentation.

**Table 1:** Study design and number of dogs that received each procedure

Time point	T0	T1	T2	T3
Days after first presentation	0	20–28	41–54	368–434
Procedure/number of dogs				
Vaccination	48	48	-	44
Clinical examination	48	48	48	44
Echocardiography	24	24	-	-
MAT	48	-	48	44
Haematology	48	-	48	-
Blood biochemistry	36	-	36	-
cTroponin I	29	-	29	-
Number of dogs receiving other vaccine components				
Nobivac® DHPPi	16	3	-	-
Nobivac® Pi	10	1	-	-

### Haematology, blood biochemistry and cTroponin I

Haematology and blood biochemistry tests and measurements of cTroponin I were performed at the Clinical Laboratory, Vetsuisse Faculty, University of Zurich. Haematology was performed from EDTA anti-coagulated whole blood within 4 h of sample collection. Blood biochemistry was determined batch-wise from serum samples stored at −80 °C. Haematology and blood biochemistry were run on a Sysmex XT-2000iV (Sysmex Corporation, Kobe, Japan) [27] and Cobas Integra 800 instrument (Roche Diagnostics AG, Rotkreuz, Switzerland), respectively. The clinical chemistry parameters included: albumin, total protein, alanine aminotransferase (ALAT), alkaline phosphatase (AP), bilirubin, urea, creatinine and lipase. The laboratory’s device-specific reference intervals were applied. cTroponin I was measured on an Immulite 1000 (Siemens Healthcare Diagnostics GmbH, Zurich, Switzerland). The serum cTroponin I concentration was measured using a commercially available assay that had been validated for the dog [28]. The test’s lower limit of detection was 0.2 ng/mL, and cTroponin I values exceeding 0.2 ng/mL were considered increased.

### Microscopic agglutination test

The MAT was performed at the National Reference Laboratory for animal leptospirosis at the Institute of Veterinary Bacteriology, Vetsuisse Faculty﻿, University of Bern, Switzerland. The MAT was performed batch-wise from serum samples stored at −80 °C that were sent by priority mail at room temperature to the laboratory. Samples were examined for the presence of antibodies against a standard panel of 14 pathogenic *Leptospira* strains commonly used in Switzerland (Table 2) by MAT according to Office International des Epizooties (OIE) standards [29]. To prevent inter-observer variability, all samples were tested by the same person. The end-point titre refers to the reciprocal of the highest serum dilution at which at least 50% agglutination occurs. In this study, sera with a titre of 100, indicating past exposure to *Leptospira* [8], were considered seropositive.

**Table 2:** The 14 *Leptospira spp.* strains used as live antigens in the microscopic agglutination test^a^

Genomospecies	Serogroup	Serovar	Strain
*Leptospira interrogans*	Australis	Australis	Ballico
	Australis	Bratislava	Jez-Bratislava
	Autumnalis	Autumnalis	Akiyami
	Bataviae	Bataviae	Swart
	Canicola	Canicola	Hond Utrecht IV
	Icterohaemorrhagiae	Icterohaemorrhagiae	RGA
	Icterohaemorrhagiae	Copenhageni	M20
	Pomona	Pomona	Pomona
	Pyrogenes	Pyrogenes	Salinem
	Sejroe	Hardjo	Hardjoprajitno
*Leptospira borgpetersenii*	Ballum	Ballum	Mus127
	Tarassovi	Tarassovi	Perepelitsin
	Sejroe	Sejroe	M84
*Leptospira kirschneri*	Grippotyphosa	Grippotyphosa	Moskva V

^a^All reference strains were obtained from the Royal Tropical Institute (KIT), Amsterdam, The Netherlands

### Echocardiography

Echocardiographic examinations were performed with continuous ECG monitoring on laterally recumbent unsedated dogs using a GE Vivid 7 ultrasound unit (Vivid 7, GE Medical Systems, Munich, Germany). Standard two-dimensional, M-mode and colour Doppler echocardiographic views were obtained from standard right parasternal views using a 7 MHz transducer. The primary focus was qualitative left ventricular size and function as well as valvular integrity and function. Quantitated parameters were left ventricular diameter in diastole and systole (LVDd and LVDs, respectively), the shortening fraction (%FS) from M-Mode images, and the left atrial to aortic ratio (LA/Ao) in short axis [30, 31].

### Statistical analyses

Statistical analyses were performed using Analyse-it® for Microsoft Excel version 1.0.5.0 (Analyse-it Software, Ltd., Leeds, United Kingdom) and GraphPad Prism 5.03 (GraphPad Software, Inc., CA, USA). The Wilcoxon signed rank test (p_w_) was used to compare the results from the same dog at two different time points. Correlation between MAT titres and time elapsed since the last *Leptospira* vaccination was assessed using the Spearman rank correlation test (correlation coefficient: r_sp_). Proportions between groups were analysed with the Fisher’s exact test (p_F_). *P*-values <0.05 were considered significant.

## Results

### Characteristics of the study population

A total of 48 dogs were included in the study. The characteristics of the study population are shown in Table 3. All but one dog had previously received a *Leptospira* vaccine; all vaccine products were inactivated bivalent *Leptospira* vaccines containing serovars from the serogroups Canicola and Icterohaemorrhagiae. The vaccines belonged to several different brands (Canigen® L (*n* = 28), Canigen® 7 (*n* = 1), Virbac, Glattbrugg, Switzerland; Canimed® L (*n* = 1), Eurican® L (*n* = 1), Merial, Lyon, France; Nobivac® Lepto (*n* = 8), Vetamun® Lepto (*n* = 6), MSD Animal Health GmbH; Vanguard® 7 (*n* = 2), Zoetis Schweiz GmbH, Zurich, Switzerland). All 48 dogs completed the primary vaccination schedule, and 44 dogs were sampled after 1 year (T3, Table 1); the remaining four dogs died within 1 year after vaccination for unrelated reasons (*n* = 2), moved to another country (*n* = 1) or did not keep the appointment after 1 year (*n* = 1). The two dogs that died were 11 and 14 years old and were euthanized because of histiocytic sarcoma or acute monoparesis that occurred 137 and 340 days after the second vaccination, respectively.

**Table 3:** Characteristics of the 48 dogs of this study

Parameter	Number of dogs	Range	Median	95% CI^a^
Age (years)	48	0.4–14	5	4.2–6.3
Body weight (kg)	48	5.5–51.2	21	18.0–25.1
Days to prior leptospirosis vaccination	47	31–1745	379	386.5–640.5
Parameter	Number of dogs (%)			
Gender				
Female intact	16 (33)			
Female castrated	16 (33)			
Male intact	14 (29)			
Male castrated	2 (4)			
Pedigree				
Yes	39 (81)			
No	9 (19)			
Breed				
Barzoi	8 (17)			
Beagle	4 (8)			
Labrador Retriever	5 (10)			
Jack Russel Terrier	2 (4)			
Rhodesian Ridgeback	2 (4)			
Shetland Sheepdog	6 (13)			
Malinois	2 (4)			
Whippet	2 (4)			
Other breeds^b^	8 (17)			

^a^CI, confidence interval

^b^Other breeds comprised: Old German Shepherd Dog, Australian Kelpie, Bavarian Mountain Hound, Golden Retriever, German Wirehaired Pointer, Small Munsterlander, Poitevin, Schipperke (each *n* = 1)

### Clinical examination

Clinical examination was unremarkable in 75–90% of the dogs at each presentation (Additional file 1). Clinical findings included vaginal discharge due to juvenile vaginitis or heat, conjunctivitis, faint heart murmurs, splenomegaly, focal pyoderma, inflammation or fistula in the anal region, red and scaly ears, fluid-filled bowel loops, enlarged prostate and depigmentation of the nose.

Following the first and second vaccination, adverse vaccine events were reported by the owners of 11 (23%) and 5 (10%) dogs, respectively (Additional file 1). The clinical signs occurred within 5 days of vaccination and were transient and mild in all cases. The clinical signs mainly included local reactions at the injection site, lethargy or inappetence. Three dogs showed a single episode of vomiting or periuria. A total of 6 out of 11 dogs with adverse events after the first vaccination had received additional vaccine components (Nobivac® DHPPi, *n* = 3, Nobivac® Pi, *n* = 3). None of the five dogs with adverse events after the second vaccination had received additional vaccine components. The proportion of animals that showed adverse vaccine events was not significantly different between dogs that had or had not received additional vaccine components (p_F_ = 1.0). Other anamnestic abnormalities that occurred after 5 days of vaccination were reported in 5 (10%) and 4 (8%) dogs after the first (T0) and second vaccination (T1), respectively. They included a single episode of vomiting or diarrhoea 10–14 days after vaccination, a depigmentation of the planum nasale that started 7 days after vaccination, acute trembling and a focal epileptic seizure 3 and 4 weeks after vaccination, respectively, coughing 3 weeks after vaccination, a reduced general condition 10 days after vaccination and tenesmus due to prostate enlargement 3 weeks after vaccination. At the time of the 1-year booster vaccination (T3), two dogs had died (see above), one dog had developed laryngeal paralysis, one dog suffered from idiopathic epilepsy and one dog had developed and recovered from an acute encephalitis.

### Microscopic agglutination test

MAT results for the 14 tested serovars are shown in Table 4 for the serogroups included in the vaccine and in Table 5 for the serogroups that are not included in the vaccine. At first presentation and before the first vaccination (T0), all dogs had negative MAT titres (< 100) for all tested serovars, except for the serovar Canicola, for which 50% of the dogs showed titres ≥100 at T0 (Table 4). All of the seropositive dogs had been previously vaccinated with a vaccine that contained a serovar of the serogroup Canicola. In the 20 dogs with titres of 100, the previous *Leptospira* vaccine had been given 101–1745 days prior to study enrolment. In the three dogs with titres of 200, the leptospirosis vaccine had been given 176–554 days prior to study enrolment, and in the one dog with a titre of 400, the vaccine had been given 129 days before enrolment. There was no significant correlation between the MAT titres to the serovar Canicola and the time elapsed since the last vaccination (r_sp_ = − 0.006).

**Table 4:** Microscopic agglutination test results for the vaccine serogroups before and after vaccination

Species	*L. interrogans*			*L. interrogans*			*L. interrogans*			*L. interrogans*			*L. interrogans*			*L. kirschneri*		
Serogroup	Canicola			Icterohaemorrhagiae			Icterohaemorrhagiae			Australis			Australis			Grippotyphosa		
Serovar	Canicola			Copenhageni			Icterohaemorrhagiae			Bratislava			Australis			Grippotyphosa		
Time point	T0	T2	T3	T0	T2	T3	T0	T2	T3	T0	T2	T3	T0	T2	T3	T0	T2	T3
Percentage of dogs with titre																		
< 100	50	0	91	100	58	95	100	98	98	100	40	98	100	11	82	100	14	95
100	**42**	**2**	**9**	0	**33**	**5**	0	**2**	**2**	0	**19**	0	0	**27**	**11**	0	**42**	**5**
200	**6**	**6**	0	0	**9**	0	0	0	0	0	**29**	**2**	0	**21**	**7**	0	**23**	0
400	**2**	**34**	0	0	0	0	0	0	0	0	**10**	0	0	**25**	0	0	**19**	0
800	0	**29**	0	0	0	0	0	0	0	0	**2**	0	0	**8**	0	0	**2**	0
1600	0	**27**	0	0	0	0	0	0	0	0	0	0	0	**6**	0	0	0	0
3200	0	**2**	0	0	0	0	0	0	0	0	0	0	0	**2**	0	0	0	0
Total number of dogs	48	48	44	48	48	44	48	48	44	48	48	44	48	48	44	48	48	44

Positive titres are shown in bold

**Table 5:** Microscopic agglutination test results for selected non-vaccine serogroups before and after vaccination

Species	*L. interrogans*			*L. interrogans*			*L. interrogans*			*L. borgpetersenii*		
Serogroup	Pomona			Autumnalis			Pyrogenes			Ballum		
Serovar	Pomona			Autumnalis			Pyrogenes			Ballum		
Time point	T0	T2	T3	T0	T2	T3	T0	T2	T3	T0	T2	T3
Percentage of dogs with titre												
< 100	100	88	87	100	42	95	100	92	82	100	100	98
100	0	**8**	**9**	0	**50**	**5**	0	**4**	**2**	0	0	**2**
200	0	**2**	**2**	0	**4**	0	0	0	0	0	0	0
400	0	**2**	**2**	0	**2**	0	0	**2**	**16**	0	0	0
800	0	0	0	0	0	0	0	**2**	0	0	0	0
1600	0	0	0	0	0	0	0	0	0	0	0	0
3200	0	0	0	0	**2**	0	0	0	0	0	0	0
Total number of dogs	48	48	44	48	48	44	48	48	44	48	48	44

Positive titres are shown in bold

Positive MAT titres (≥ 100) measured after the second vaccination (T2) were most common for the serovar Canicola (100%), followed by the serovars Australis (89%), Grippotyphosa (86%), Bratislava (60%), Autumnalis (58%), Copenhageni (42%), Pomona (12%), Pyrogenes (8%) and Icterohaemorrhagiae (2%, Tables 4 and 5). Median to high titres (≥ 400) were most commonly found for the serovar Canicola (92%), followed by the serovars Australis (41%), Grippotyphosa (21%), Bratislava (12%), Autumnalis (4%), Pyrogenes (4%) and Pomona (2%); none of the dogs developed titres ≥400 for the serovars Copenhageni or Icterohaemorrhagiae. Maximal MAT titres measured at T2 for the serovars were as follows: Canicola (3200), Australis (3200), Grippotyphosa (800), Bratislava (800), Autumnalis (3200), Copenhageni (200), Pomona (400), Pyrogenes (800) and Icterohaemorrhagiae (100).

At the 1-year booster appointment (T3), 2–18% of the dogs showed positive MAT titres to serovars of the vaccine serogroups, and the titres were generally low (< 400, Table 4). A total of 2–18% of the dogs showed positive MAT titres to the non-vaccine serovars Pomona, Autumnalis, Pyrogenes and Ballum (Table 5). The seven dogs with an MAT titre of 400 against the serovar Pyrogenes at T3 had negative titres against the other tested serovars at this time point and had also been seronegative for the serovar Pyrogenes following the second vaccination (T2). Positive MAT titres were not detected at any time point during the study for *L. interrogans* serovar Bataviae or for *L. borgpetersenii* serovars Tarassovi, Hardjo and Sejroe (data not shown).

### Haematology, blood biochemistry and cTroponin I

The haematological parameters determined before (T0) and after the second vaccination (T2) revealed no significant differences, with the exception of a slight but significant increase in haematocrit (p_W_ = 0.02) and a decrease in total monocyte count at T2 compared to T0 (p_W_ = 0.04; Additional file 2). For the blood biochemistry results, significant differences between T0 and T2 were only evident for the AP activity (decrease; p_W_ = 0.04) and the urea concentration (increase; p_W_ = 0.03; Additional file 3). These differences were small, and the median AP activity and urea concentration remained within the reference range at both time points. The cTroponin I concentrations were obtained in 29 dogs. At T0, cTroponin I concentrations were below the detection limit in 27 dogs; two dogs (both Barzois) had measurable cTroponin I levels of 0.39 and 0.50 ng/mL. At T2, cTroponin I concentrations were below the detection limit in 27 dogs; two dogs (both Barzois) had measurable cTroponin I levels of 0.23 and 0.29 ng/mL; both dogs had had undetectable values at T0. There was no significant difference between the cTroponin I levels at T0 and T2.

### Echocardiography

Left ventricular (LVDd) and left atrial (LA/Ao) size did not significantly change between the first (T0) and the second (T1) vaccination, and mitral insufficiency could not be documented in any dog. Additionally, systolic function, as assessed by LVDs and %FS, did not change significantly between T0 and T1. Looking at individual dogs, there was not any dog that showed a notable increase in LVDd or LVDs. In none of the dogs could any arrhythmia be recognized at any time point during the study.

## Discussion

This is the first independent study evaluating the serological and clinical response in field dogs following vaccination with a novel tetravalent *Leptospira* vaccine. Our results suggest that the vaccine was generally well tolerated and that there was marked variation among individual dogs and among different vaccine serovars regarding the extent of seroconversion following vaccination.

Up to 23% of the dogs in this study showed clinical signs following vaccination, but they were mild and transient in all cases and did not include anaphylactoid reactions. *Leptospira* vaccines have been said to carry an increased risk of adverse events in puppies younger than 12 weeks of age and in small-breed dogs [32], but a recent study evaluating the incidence rate of owner-reported post-vaccination adverse events found no significant increase in hypersensitivity reactions in dogs vaccinated against *Leptospira* compared with other vaccines [25]. In the present study, most of the adverse vaccine events included discomfort at the injection site and transient lethargy and inappetence. These are common clinical signs induced by vaccination and have also been reported for other vaccine products [33]. Because additional vaccine components were administered in six of the eleven dogs that showed clinical signs after the first vaccination, the clinical signs could also have been attributable to other vaccine components. However, there was no significant difference in the frequency of adverse events between dogs that did or did not receive other vaccine components at the first or second vaccination. In this study, we decided to administer additional vaccine components as indicated to mimic the situation in the field, where *Leptospira* vaccines are usually administered together with other vaccine components registered for concurrent application. In light of the high incidence and mortality rate of clinical leptospirosis in dogs in Europe [4, 5, 16], the benefit of vaccinating dogs with this novel tetravalent vaccine highly outweighs the risk of adverse events following vaccination.

The highest MAT titres after vaccination for the tested serovars were observed for the serovars Canicola, Australis, Grippotyphosa, Bratislava and Autumnalis; the MAT titres were generally low (up to 200) for the vaccine serovar Copenhageni (serogroup Icterohaemorrhagiae), with only 42% of the study population showing positive titres after the second vaccination. One recent study evaluated vaccine-associated *Leptospira* antibodies in client-owned dogs following vaccination with four different tetravalent *Leptospira* vaccines (all containing the serovars Canicola, Grippotyphosa, Icterohaemorrhagiae and Pomona) [10]. The authors found a comparable percentage of dogs with positive titres for the serovars Canicola (100%), Grippotyphosa (72%) and Bratislava (44%) after vaccination as in the present study, but in contrast to our results, 94% of the dogs developed positive titres to the serovar Icterohaemorrhagiae. The four vaccines tested in the previous study were different from the vaccine used in the present study; different vaccines could vary in their potential to induce seroconversion to vaccine serovars depending on the strains used in the vaccines. Alternatively, natural exposure or differences in immune status could have affected the vaccine responses of individual dogs in our study. Accordingly, we observed highly variable vaccine responses among individual dogs, as also reported in previous studies [10]. It could be argued that we missed the maximal MAT titres against the Icterohaemorrhagiae serogroup, because we sampled the dogs only once 16–31 days after the second immunization, whereas a previous study followed the MAT titres for several weeks following immunization [10]. However, Martin et al. found the highest MAT titres for the vaccine serovars at weeks 4 and 7 after the start of immunization, which is similar to the time point used in the present study to evaluate seroconversion following vaccination. Importantly, a lack of seroconversion does not imply lack of immune protection against the vaccine serovar Copenhageni. Evaluating the protective potential of *Leptospira* vaccines by use of MAT titres does not provide the entire picture, because previous studies have shown that post-vaccination titres may not correlate with protection [11, 34].

Seroconversion following vaccination was also observed for some non-vaccine serovars, including Autumnalis, Pomona and Pyrogenes. This finding is not unexpected and can be explained by cross-reactivity in MAT among different *Leptospira* serovars. MAT titres are not serovar-specific, and also in recent studies, dogs developed positive titres against non-vaccine serovars following vaccination with bi- and tetravalent *Leptospira* vaccines [10–12]. In one study, dogs showed even highest titres to non-vaccine serovars [12]. In the present study, one dog showed maximal seroconversion for the non-vaccine serovar Autumnalis following vaccination, with a titre of 3200 in MAT, although the titres for all of the vaccine serovars were negative at that time except for the serovar Canicola (800). Besides vaccination, natural exposure to the serovar Autumnalis coinciding with vaccination could have accounted for seroconversion in this animal. However, the dog showed no clinical signs or abnormalities in haematology and blood biochemistry compatible with leptospirosis at that time. Our results indicate that even high titres (≥ 800) against non-vaccine serovars should be interpreted with caution in vaccinated dogs exhibiting signs consistent with leptospirosis.

Half of the dogs showed positive MAT titres at study enrolment for the serovar Canicola, although vaccines containing this serovar had been administered to the dogs up to 1745 days prior to sampling. The dog is assumed to be a natural reservoir host of *L. interrogans* serovar Canicola, and before the introduction of efficient vaccines, clinical leptospirosis in dogs has commonly been associated with this serovar. Because the present study was performed in privately owned dogs with outdoor access, natural exposure to leptospires in addition to vaccination could have resulted in antigenic stimulation in these dogs. Our results are in contrast to an earlier study that evaluated *Leptospira* antibodies in privately owned dogs in Switzerland between 1991 and 1996 [35]. In that study, 34% of the dogs showed positive *Leptospira* antibody titres, with seroconversion to other serovars, e.g., Bratislava, Australis, Icterohaemorrhagiae, Pomona and Grippotyphosa, being common. Moreover, in a survey of 259 dogs from Switzerland over a 10-year-period before the introduction of the tetravalent vaccine, only 12.3% of the dogs showed seropositivity to serovar Canicola [5]. We cannot conclude from the present data whether the positive MAT titres to Canicola were due to previous vaccination or natural exposure to this serovar. However, in light of the large time span elapsed since the last vaccination in some of the seropositive dogs, natural exposure seems likely, and vaccination against this serovar can still be advocated.

In the present study, dogs were specifically examined for evidence of myocarditis due to anecdotal reports of cardiac changes after vaccination with this new vaccine. There is some credibility to such a potential association, because natural infection with leptospirosis may, indeed, cause myocarditis. Even though clinically important myocarditis is rare in dogs and people after natural infection [6, 36], subclinical myocarditis may be quite commonly present in both species based on biochemical abnormalities, specifically the elevation of cTroponin I [6], and histopathological findings [7]. Furthermore, myocarditis has been documented as an adverse event following vaccination in humans, such as after smallpox vaccination [37]. The present study found no echocardiographic, electrocardiographic or biochemical evidence of subclinical vaccine-induced myocarditis. Based on the limited number of dogs included in the present study, myocarditis as a rare complication of leptospirosis vaccination cannot, however, completely be ruled out.

The present study has some limitations. Due to the prospective study design, the number of dogs included in the study was too small to evaluate rare adverse events induced by the vaccine. This could be assessed by large retrospective studies and pharmacovigilance data. The present study also did not follow the course of the MAT titres after vaccination and therefore cannot evaluate the time point of maximal MAT titres and the duration of seropositivity induced by the vaccine. Third, the inclusion of other vaccine components could have influenced the clinical and serological response of the dogs. However, we chose this setting to most accurately mimic the situation in the field, where *Leptospira* vaccines are commonly administered together with other vaccine components licenced for concurrent administration. Finally, measurements of cTroponin I were only obtained before the first and 16–31 days after the second vaccination. It can be argued that an elevation might have been present earlier after vaccination. However, the complete absence of any evidence of morphological, functional and electrical cardiac abnormalities makes clinically important myocardial damage very unlikely.

## Conclusions

The present study indicates that the novel tetravalent *Leptospira* vaccine Nobivac® L4 is generally well-tolerated and that clinical signs following vaccination were mild and transient. Seroconversion following vaccination differed considerably among individual dogs and among the vaccine serovars. High titres to non-vaccine serovars were present in some dogs following vaccination, which hampers the interpretation of single MAT titres in vaccinated dogs with clinical signs consistent with leptospirosis. In the light of the high incidence and mortality rate associated with clinical leptospirosis in dogs in Europe, vaccination with this tetravalent vaccine can be advocated for every dog with potential exposure to leptospires.

## Additional files

Additional file 1: Medical history and clinical examination findings at different time points before and after vaccination. (DOCX 37 kb)

Additional file 2: Selected haematology results of the dogs before and after vaccination. Values outside the reference range are shown in bold, significant differences between T0 and T2 are shown in italics. (DOCX 35 kb)

Additional file 3: Selected blood biochemistry results before and after vaccination. Values outside the reference range are shown in bold, significant differences between T0 and T2 are shown in italics. (DOCX 35 kb)

## Abbreviations

ALAT: Alanine aminotransferase
AP: Alkaline phosphatase
CI: Confidence interval
cTroponin I: Cardiac Troponin I
%FS: Fractional shortening (%)
MAT: Microscopic agglutination test
LA/Ao: Left atrial to aortic ratio
LVDd: Left ventricular diameter in diastole
LVDs: Left ventricular diameter in systole
OIE: Office International des Epizooties
p_F_: *p*-value of Fisher’s exact test
p_w_: *p*-value of Wilcoxon signed rank test
r_sp_: Correlation coefficient of Spearman rank correlation test
T0: Before first vaccination
T1: Second vaccination 20–28 days after the first vaccination
T2: 41–54 days after the first and 16–31 days after the second vaccination
T3: 368–434 days after the first vaccination and 347–413 days after the second vaccination (1 year booster)
