# Supplementary material for: Clinical, serological and echocardiographic examination of healthy field dogs before and after vaccination with a commercial tetravalent leptospirosis vaccine
Source: BMC Vet Res. 2017 May 25;13:138. doi: 10.1186/s12917-017-1056-x (PMC5445508; doi:10.1186/s12917-017-1056-x)
Supplement: Supplementary file 1 — Medical history and clinical examination findings at different time points before and after vaccination. (DOCX 37 kb) [file 12917_2017_1056_MOESM1_ESM.docx]

#### Additional file 1. Medical history and clinical examination findings at different time points before and after vaccination.

| **Time point** | **T0** | | **T1** | | **T2** | | **T3** |  |
| --- | --- | --- | --- | --- | --- | --- | --- | --- |
|  | **n (%)** | **Details (n)** | **n (%)** | **Details (n)** | **n (%)** | **Details (n)** | **n (%)** | **Details (n)** |
| **Medical history** |  |  |  |  |  |  |  |  |
| Unremarkable | 48 (100) | - | 32 (67) | - | 39 (81) | - | 37 (84) | - |
| Adverse vaccine events^1^ | n.a.^2^ | - | 11 (23)^3^ | Local discomfort (4) or swelling (1) at the injection site, transient lameness (1), vomiting (2), periuria (1), reduced appetite (1), reduced general condition (2) | 5 (11) | Local discomfort at the injection site (3), transient lameness (1), reduced general condition (1) | n.a.^2^ | - |
| Other abnormalities | 0 (0) | - | 5 (10) | Vomiting (1), diarrhoea (2), depigmentation planum nasale (1), panting and trembling (1) | 4 (8) | Coughing (1), focal epileptic seizure (1), reduced general condition (1), tenesmus (1) | 7 (16) | Laryngeal paralysis (1), epilepsy (1), encephalitis (1), anal inflammation (1), corneal ulceration (1), dermal alterations (2) |
| **Clinical examination** |  |  |  |  |  |  |  |  |
| Unremarkable | 43 (90) | - | 36 (75) |  | 36 (75) | - | 35 (80) | - |
| Clinical findings^4^ | 5 (10) | Vaginal discharge (1), splenomegaly (1), conjunctivitis (1), anal inflammation (1), heart murmur (2) | 12 (25) | Conjunctivitis (6), vaginal discharge (3), focal pyoderma (1), anal inflammation (1), nose depigmentation (1), heart murmur (1) | 12 (25) | Conjunctivitis (8), enlarged prostate (1), fluid-filled bowel loops (1), anal inflammation (1), nose depigmentation (1), heart murmur (1) | 9 (20) | Conjunctivitis (4), anal fistula (1), vaginal discharge (1), red and scaly ears (1), heart murmur (3) |

^1^ Adverse vaccine events based on clinical signs and occurrence within ≤ 5 days of vaccination. For details see text. ^2^ n.a., not applicable. ^3^ One dog showed two clinical signs compatible with an adverse vaccine event (swelling at the injection site and apathy). ^4^ One dog showed two clinical signs (anal inflammation and conjunctivitis) at each time point.
