# Supplementary material for: Clinical, serological and echocardiographic examination of healthy field dogs before and after vaccination with a commercial tetravalent leptospirosis vaccine
Source: BMC Vet Res. 2017 May 25;13:138. doi: 10.1186/s12917-017-1056-x (PMC5445508; doi:10.1186/s12917-017-1056-x)
Supplement: Supplementary file 2 — Selected haematology results of the dogs before and after vaccination. Values outside the reference range are shown in bold, significant differences between T0 and T2 are shown in italics. (DOCX 35 kb) [file 12917_2017_1056_MOESM2_ESM.docx]

#### Additional file 2: Selected haematology results of the dogs before and after vaccination. Values outside the reference range are shown in bold, significant differences between T0 and T2 are shown in italics.

| **Haematology parameter** |  | **Haematocrit (%)** | |  | **Leuk^1^ (**×**10^3^/μl)** | |  | **Plat^2^ (**×**10^3^/μl)** | |  | **Neutro^3^ (10^3^/μl)** | |  | **Lymph^4^ (**×**10^3^/μl)** | |  | **Eos^5^ (**×**103/μl)** | |  | **Mono^6^ (**×**10^3^/μl)** | |
| --- | --- | --- | --- | --- | --- | --- | --- | --- | --- | --- | --- | --- | --- | --- | --- | --- | --- | --- | --- | --- | --- |
| **Time point** |  | **T0** | **T2** |  | **T0** | **T2** |  | **T0** | **T2** |  | **T0** | **T2** |  | **T0** | **T2** |  | **T0** | **T2** |  | **T0** | **T2** |
| **Median** |  | *46.0***^7^** | *47.1***^7^** |  | 7.9 | 7.8 |  | 257 | 254 |  | 4.6 | 4.3 |  | 2.3 | 2.4 |  | 0.46 | 0.44 |  | *0.34^8^* | *0.33^8^* |
| **Minimum** |  | **39.4** | **40.4** |  | **3.7** | **4.33** |  | **121** | 165 |  | 2.5 | 2.9 |  | **1.0** | **0.9** |  | 0.11 | 0.14 |  | **0.12** | **0.12** |
| **Maximum** |  | **59.4** | **56.0** |  | **16.7** | **13.4** |  | **411** | **444** |  | **10.8** | **8.5** |  | **4.6** | **4.2** |  | 1.21 | **2.23** |  | 0.88 | 0.77 |
| **Reference range** |  | 42 - 55 | |  | 4.7 - 11.3 | |  | 130 - 394 | |  | 2.5 - 7.4 | |  | 1.2 - 3.4 | |  | 0.12 - 1.29 | |  | 0.20 - 0.92 | |
| **Total number of dogs** |  | 48 | |  | 48 | |  | 47 | |  | 48 | |  | 48 | |  | 48 | |  | 48 | |

^1^Leuk, leukocytes, ^2^Plat, platelets, ^3^Neutro, neutrophils, ^4^Lymph, lymphocytes; ^5^Eos, eosinophils, ^6^Mono, monocytes, ^7^ Significant difference with p_W_= 0.02, ^8^ Significant difference with p_W_= 0.04.
