# Supplementary material for: Clinical, serological and echocardiographic examination of healthy field dogs before and after vaccination with a commercial tetravalent leptospirosis vaccine
Source: BMC Vet Res. 2017 May 25;13:138. doi: 10.1186/s12917-017-1056-x (PMC5445508; doi:10.1186/s12917-017-1056-x)
Supplement: Supplementary file 3 — Selected blood biochemistry results before and after vaccination. Values outside the reference range are shown in bold, significant differences between T0 and T2 are shown in italics. (DOCX 35 kb) [file 12917_2017_1056_MOESM3_ESM.docx]

#### Additional file 3: Selected blood biochemistry results before and after vaccination. Values outside the reference range are shown in bold, significant differences between T0 and T2 are shown in italics.

| **Biochemistry parameter** | **Alb^1^ (g/L)** | |  | **TP^2^ (g/L)** | |  | **ALAT^3^ (U/L)** | |  | **AP^4^ (U/L)** | |  | **Bil^5^ (μmol/L)** | |  | **Urea (mmol/L)** | |  | **Crea^6^ (μmol/L)** | |  | **Lipase (U/L)** | |
| --- | --- | --- | --- | --- | --- | --- | --- | --- | --- | --- | --- | --- | --- | --- | --- | --- | --- | --- | --- | --- | --- | --- | --- |
| **Time point** | T0 | T2 |  | T0 | T2 |  | T0 | T2 |  | T0 | T2 |  | T0 | T2 |  | T0 | T2 |  | T0 | T2 |  | T0 | T2 |
| **Median** | 37.0 | 36.9 |  | 56.2 | 54.3 |  | 35.2 | 41.0 |  | *37.0^7^* | *34.0^7^* |  | < 2.5 | < 2.5 |  | *5.9^8^* | *6.6^8^* |  | 81.5 | 82.5 |  | 14.1 | 27.6 |
| **Minimum** | 32.3 | **16.2** |  | **46.2** | **20.8** |  | 22.4 | 22.2 |  | **7.2** | **7.1** |  | < 2.5 | < 2.5 |  | 4.0 | **3.2** |  | 55.1 | **28.3** |  | **9.8** | **9.3** |
| **Maximum** | **44.0** | **41.2** |  | 67.4 | 67.8 |  | **90.1** | **125.8** |  | **104.8** | 98.0 |  | 2.8 | < 2.5 |  | 8.7 | **12.3** |  | **122.0** | **122.3** |  | **701.6** | **178** |
| **Reference range** | 29 - 37 | |  | 56 - 71 | |  | 20 - 93 | |  | 20 - 98 | |  | < 2.5 | |  | 3.8 - 9.4 | |  | 50 - 119 | |  | 24 - 108 | |
| **Number of dogs** | 36 | |  | 36 | |  | 36 | |  | 36 | |  | 33 | |  | 36 | |  | 36 | |  | 30 | |

^1^Alb, albumin, ^2^TP, total protein, ^3^ALAT, alanine aminotransferase, ^4^AP, alkaline phosphatase, ^5^Bil, bilirubin; ^6^Crea, creatinine, ^7^ significant difference with p_W_= 0.04, ^8^ significant difference with p_W_= 0.03.
